# Comparison of Preoperative Refractive Status and Postoperative Outcomes Following Transepithelial Photorefractive Keratectomy

**DOI:** 10.3390/life16060997

**Published:** 2026-06-13

**Authors:** Jiunn-Liang Chen, Kai-Ling Peng

**Affiliations:** 1 Department of Ophthalmology, Kaohsiung Veterans General Hospital, 386, Ta-Chung 1st Road, Kaohsiung 813, Taiwan; eyegogo@gmail.com; 2 Department of Optometry, Shu-Zen Junior College of Medicine and Management, Kaohsiung 821, Taiwan

**Keywords:** photorefractive keratectomy, refraction, myopia, astigmatism, central corneal thickness

## Abstract

Transepithelial photorefractive keratectomy (Trans-PRK) offers superior re-epithelialization and visual recovery. This study evaluates the impact of preoperative refractive status on clinical outcomes and identifies prognostic factors across varying myopic severities. This retrospective observational study included 125 eyes [64 patients; age > 20 years; best-corrected visual acuity (BCVA) ≥ 20/25] that underwent Trans-PRK between March and December 2022. Patients were stratified into low myopia (LM: > −5.0 D), moderate-to-high myopia (MHM: −5.0 D to −8.0 D), and extremely high myopia (EHM: ≤ −8.0 D) groups. Analysis focused on preoperative refraction, intraoperative parameters, postoperative uncorrected visual acuity (UCVA), and corneal conditions of superficial punctate keratitis (SPKs) and haze. The mean age was 30.20 ± 6.34 years, with a mean initial manifest sphere (MS) of −6.42 ± 2.27 diopter (D) overall and −3.73 ± 0.15 D, −6.28 ± 0.13 D, and −9.17 ± 0.15 D in the LM, MHM, and EHM groups, respectively. At a mean follow-up of 6.69 ± 3.73 months, the overall mean final manifest spherical equivalent (MSE) was −0.12 ± 0.73 D, and the mean final UCVA was 0.01 [Snellen equivalent (SE), 205/200] ± 0.08 logMAR. Predictability was 94.4%, 88.88%, and 94.3% for the final MS ≤ −1.0 D, final MSE ≤ −1.0 D, and UCVA 0.8, respectively. In the LM and MHM groups, cycloplegic and subjective refractions showed the highest concordance with emmetropia, whereas initial manifest refractions were most accurate for the EHM group. Corneal SPK incidence declined from 32.2% (1 month) to 1.6% (6 months), primarily localized to EHM eyes. Corneal haze peaked at 28.2% at three months before receding to 9.4% by 6 months. Refractive and visual stability were achieved by the third month for the LM and MHM groups, whereas the EHM group (mean MSE: −9.59 ± 0.15 D) required six months to reach both refractive and visual plateaus. Despite transiently higher rates of corneal SPKs and haze in EHM eyes, final visual outcomes remained excellent, achieving a mean UCVA of 18/20.

## 1. Introduction

In 2020, myopia, the leading cause of distance vision impairment, affected 34.0% of the global population, a figure projected to rise to 39.9% by 2030 [1]. The prevalence of myopia was 2620 million in 2020 [1], and 800,000 to 1.4 million corneal refractive surgeries are performed annually in the United States, including photorefractive keratectomy (PRK), laser in situ keratomileusis (LASIK), and small incision lenticular extraction (SMILE) [2]. Although all three refractive surgeries are now safer and more precise than ever, distinct limitations persist: femtosecond LASIK (FS-LASIK) remains subject to flap-associated complications despite replacing the microkeratome; single-step transepithelial PRK (Trans-PRK) accelerates recovery and offers high revisability but faces epithelial healing challenges in extreme high myopia [3,4,5]; and SMILE, despite offering superior biomechanical stability to both alternative procedures, typically relies on secondary surface ablation for enhancement [6] instead of repeated lenticule extraction.

In 1996, PRK became the first form of refractive surgery to use the excimer laser for surface ablation [7,8]. Conventional PRK utilizes manual or alcohol-assisted epithelial removal, which may cause basement membrane injury, alcohol toxicity, and delayed healing. These methods are frequently associated with significant pain and stromal haze, even with adjunctive Mitomycin-C (MMC) [9]. Introduced in 2007, the current generation of Trans-PRK performs a single-step ablation of both the epithelium and stroma [10], replacing earlier two-step procedures that used phototherapeutic keratectomy (PTK) to remove the epithelium first [11,12,13]. Ablative spot geometry, first reported in 2017, creates a smoother surface profile that promotes faster corneal re-epithelialization, enhances visual recovery, and mitigates early postoperative pain [3,4].

In this study, we aim to compare preoperative initial manifest, cycloplegic, and subjective refractions to identify which measurement achieves a postoperative manifest refraction closest to emmetropia following Trans-PRK. The novelty of this approach lies in directly isolating these distinct preoperative refraction modalities to establish the most reliable baseline for maximizing predictability, particularly in highly challenged eyes. We primarily hypothesize that anchoring laser programming on cycloplegic refractions yields the most stable long-term refractive predictability and minimizes the risk of late-stage regression.

## 2. Methods

### 2.1. Patients

This retrospective, observational study examined eyes that underwent Trans-PRK (SCHWIND AMARIS 750S excimer laser, SCHWIND eye-tech-solutions GmbH & Co. KG, Kleinostheim, Germany) between March 2022 and December 2022 at the Kaohsiung Veterans General Hospital. All patients signed informed consent following the tenets of the Declaration of Helsinki. The study was reported in accordance with the Strengthening the Reporting of Observational Studies in Epidemiology (STROBE) statement guidelines and approved by the institutional ethical board committee. The inclusion criteria for the study were as follows: age older than 20 years, eyes with best-corrected visual acuity (BCVA) better than 16/20, discontinuation of soft contact lens use for more than 2 weeks before treatment, a predicted postoperative residual stromal bed thickness exceeding 300 µm, and a minimum postoperative follow-up duration of one month. The exclusion criteria were as follows: eyes that had ocular surface diseases, including corneal diseases, previous refractive surgery with residual myopia requiring revision, presbyopia requiring myopia treatment, corneal or lens opacity, corneal trauma with primary repair, prior cornea or lens surgeries, glaucoma history with medication and surgeries, and retinal or foveal diseases with surgery history. A total of 125 eyes from 64 consecutive patients were included in the study.

### 2.2. Preoperative Factors

Preoperative evaluation of refractions [sphere, cylinder, and spherical equivalent (SE)] included initial manifest, cycloplegic, and subjective refractions. Subjective refraction was performed to achieve clear vision with balanced red—green light duo-chrome testing. The determined refraction for laser programming was selected by the surgeon based on baseline manifest, cycloplegic, and subjective refractions, while accounting for corneal profiles to ensure a predicted postoperative residual stromal bed thickness of more than 300 µm. BCVA was assessed using trial frames after a two-week soft contact lens holiday. The other recorded information included age, gender, central corneal epithelial thickness (CCET), and CCT. The data of both CCET and CCT were measured via anterior segment optical coherence tomography (AS-OCT).

Patients were stratified into three groups based on initial manifest sphere (MS): extremely high myopia (EHM; ≤−8.0 D), moderate-to-high myopia (MHM; −5.0 D to −8.0 D), and low myopia (LM; >−5.0 D).

### 2.3. Intraoperative Factors

During surgeries, the recorded data included determined refraction, performed and residual CCT, measured and performed CCET, mitomycin (MMC) soaking duration, ablation zone diameter around 6.0–6.5 mm, and laser application interval. We further analyzed the related factors of intraoperative factors, including differences between the measured and performed CCET, to determine if they affected final corneal presentation, manifest refraction, and uncorrected visual acuity (UCVA).

### 2.4. Surgical Technique

All surgeries were performed using a sixth-generation SCHWIND AMARIS 750S excimer laser. Treatments were planned based on the performed refraction with an aberration-neural profile and proper ablation zone according to patients’ profiles calculated using the ORK-CAM software module (Software Version: 6.1.2117.705, SCHWIND eye-tech solutions GmbH & Co. KG, Kleinostheim, Germany).

Following topical anesthesia (0.5% proparacaine) and standard disinfection, transepithelial laser ablation was performed after verifying the correct horizontal and torsional eye positions on the computer and with active eye tracking. After laser ablation, 0.02% MMC was applied via a surgical sponge to the stromal bed for 60 s, followed by chilled balanced salt solution irrigation. A bandage contact lens (BCL; Acuvue Oasys, Johnson & Johnson Vision, Jacksonville, FL, USA) was applied, and topical 1% Pred Forte, 0.5% Cravit, 0.1% Nevanac, and autologous serum were initiated. Following complete re-epithelialization and BCL removal, the postoperative topical steroid regimen was transitioned to 0.3% betamethasone/neomycin solution (Rinderon-A) three times daily during the second month, followed by 0.1% Fluorometholone twice daily throughout the third month.

### 2.5. Postoperative Factors

Postoperative visits assessed manifest refraction, UCVA, and corneal status, specifically evaluating the presence or absence of superficial punctate keratitis (SPK) and corneal haze. UCVA values were converted to the logarithm of the minimum angle of resolution (logMAR) for analysis.

### 2.6. Statistical Analysis

We compared preoperative refractive metrics to determine refractions using Linear Mixed Models (LMMs). To account for inter-eye dependency (bilateral data), an LMM was used for continuous outcomes (SE and logMAR), while Generalized Estimating Equations (GEEs) were utilized for binary outcomes (haze and SPK). The results are reported as regression coefficients (*B*), Odds Ratios (OR), and 95% Confidence Intervals (95% CI). Post hoc pairwise comparisons with Bonferroni correction were applied among myopia groups. Data were analyzed using the IBM SPSS version 31.0 (Armonk, NY, USA). *p* < 0.05 denotes statistical significance.

## 3. Results

One hundred and twenty-five eyes of sixty-four patients, comprising twenty-one men (33.6%) and forty-three women (66.4%), underwent Trans-PRK during the study period. The mean age was 30.20 ± 6.34 years (range, 20–47; median, 29), and the mean follow-up was 6.69 ± 3.73 months (range, 1–13.96 months; median, 6.29). Among the treated eyes, 28% (35 eyes) were in the LM group, while 42% (53 eyes) were in the MHM group and 30% (37 eyes) were in the EHM group. The mean manifest sphere (MS) power was −6.42 ± 2.27 D in the total sample, −3.73 ± 0.15 D in the LM group, −6.28 ± 0.13 D in the MHM group and −9.17 ± 0.15 D in the EHM group while the mean manifest spherical equivalent (MSE) was −7.16 ± 2.31 D in the total sample, −4.69 ± 0.20 D in the LM group, −7.09 ± 0.16 D in the MHM group and −9.59 ± 0.20 D in the EHM group. The mean final MS was 0.17 ± 0.71 D in the total sample, 0.33 ± 0.13 D in the LM group, 0.23 ± 0.11 D in the MHM group and −0.07 ± 0.13 D in the EHM group while the mean final MSE was −0.12 ± 0.73 D in the total sample, 0.05 ± 0.13 D in the LM group, −0.05 ± 0.11 D in the MHM and −0.38 ± 0.13 D in the EHM group. The mean final UCVA was 0.01 [Snellen equivalent (SE), 205/200] ± 0.08 logMAR in the total sample, 0.00 (SE, 200/200) ± 0.01 logMAR in the LM group, 0.01 (SE, 205/200) ± 0.01 logMAR in the MHM group, and 0.01 (SE, 205/200) ± 0.02 logMAR in the EHM group, with no significant difference between the three groups. Table 1 summarizes the baseline characteristics of the study population. Furthermore, the final MS ≤ −1.0 D accounted for 100% in the LM, 94.3% in the MHM group, 89.2% in the EHM group, and 94.4% in the total sample, while SE ≤ −1.0 D accounted for 97.1% in the LM group, 88.7% in the MHM group, 81.1% in the EHM group, and 88.88% in the total sample.

### 3.1. The Related Factors Affecting the Final MSE and the Refractive Changes After Trans-PRK

An LMM was conducted to account for the correlation between eyes of the same patient, and sex was identified as a consistent and significant predictor of final MSE across all multivariate iterations (*p* = 0.002). Male patients showed final hyperopia (0.22 ± 0.13 D) while female patients tended toward final myopia (−0.29 ± 0.09 D). CCT (*p* = 0.011) and the presence of final corneal haze (*p* = 0.043) were also significant independent factors influencing the final refractive outcome. Final UCVA was a significant predictor of the final MSE (*p* = 0.009), confirming the close relationship between refractive accuracy and functional visual outcomes (Table 1). However, the LMM analysis demonstrated high refractive precision with a final mean MSE in total near emmetropia (*p*= 0.880). While lacking formal statistical significance, the interaction between final corneal SPKs and final UCVA suggested a potential role for surface regularity in refractive outcomes (*p*= 0.060).

#### 3.1.1. Spherical Power

**Figure 1:**
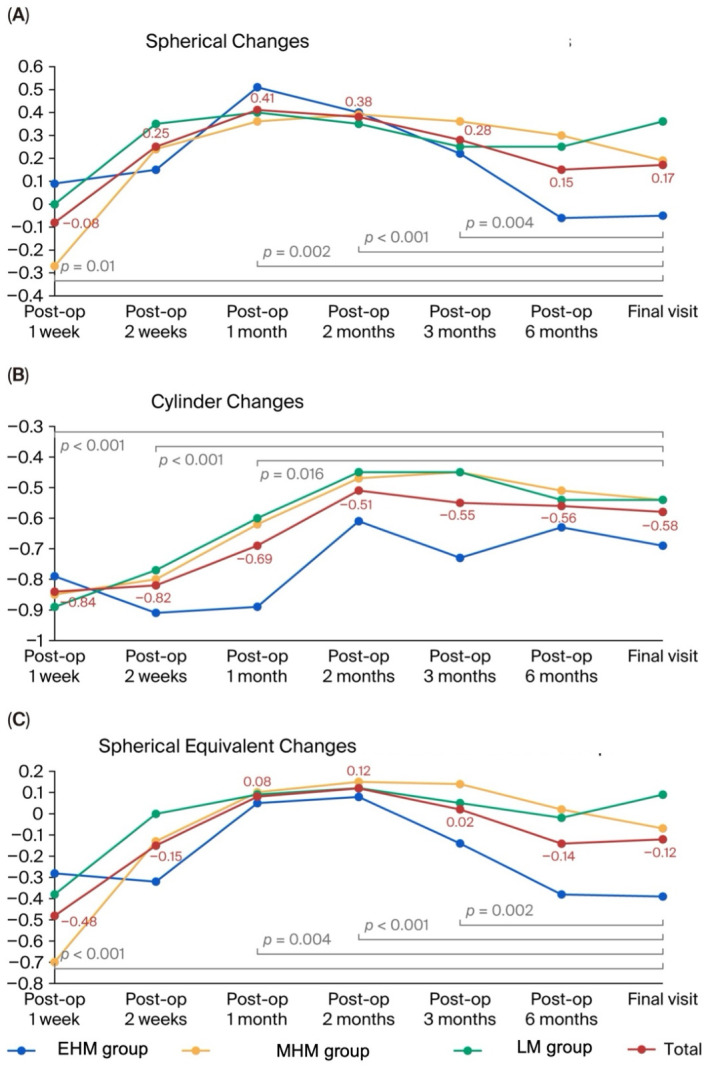
(**A**) Regarding spherical changes, all three groups of eyes experienced hyperopia shift within one month postoperatively. Between one and six months postoperatively, the extremely high myopia (EHM) eyes experienced the largest and fastest myopia shift, while the moderate-to-high myopia (MHM) eyes showed a relatively mild myopia shift, and the low myopia (LM) eyes showed the least myopia shift, but both groups remained hyperopia. After six months postoperatively, the EHM eyes remained myopic while the MHM eyes developed mildly myopic changes within a hyperopic state, and the LM group further progressed more hyperopia. Overall, the spherical powers did not differ significantly from the final values six months postoperatively. (**B**) The LM and MHM groups followed a similar trend, characterized by a linear decrease in astigmatism through the third month, followed by a slight increase. The EHM group conversely exhibited a continuous decline in astigmatism from the second month through the final follow-up. Overall, cylinder power achieved statistical stability relative to final outcomes by two months postoperatively. (**C**) Regarding changes in spherical equivalent, all three groups of eyes progressed to hyperopia by two months postoperatively. The EHM group subsequently reversed to myopia, the MHM group had a slight reduction but remained hyperopia, and the LM group maintained a similar extent of hyperopia throughout. Overall, the manifest spherical equivalent values did not exhibit significant differences from the final values at six months postoperatively.

Overall, mean spherical power shifted from −0.09 ± 0.38 D (1 week) to a peak hyperopic shift of 0.41 ± 0.71 D (1 month) before regressing toward emmetropia (0.17 ± 0.71 D at final visit; Figure 1A). Spherical power achieved statistical stability relative to the final refraction by the six-month mark. Postoperatively, all groups exhibited a hyperopic shift within the first month. The EHM group experienced a significant myopic regression, between one and six months, ultimately remaining in a myopic state. In contrast, the LM and MHM groups maintained a hyperopic state, though the MHM group showed mild myopic regression while the LM group trended toward further hyperopia.

#### 3.1.2. Cylinder Power

The total population showed a gradual reduction in mean cylinder power, stabilizing by the second month postoperatively (−0.58 ± 0.43 D at final visit; Figure 1B). The LM and MHM groups demonstrated rapid astigmatic reduction, stabilizing within two months. The EHM group exhibited an initial transient increase in cylinder power during the first two weeks but reached stability by two months, maintaining consistently higher residual astigmatism than the other groups.

#### 3.1.3. Manifest Spherical Equivalent (MSE)

In the total sample, the mean MSE trended from −0.48 ± 0.80 D (1 week) to a stable final value of −0.12 ± 0.73 D (Figure 1C). Significant differences in the final MSE in the overall cohort persisted throughout the first three months (*p* < 0.001). The LM and MHM groups reached stability relative to the final visit by one month. Conversely, the EHM group showed a peak hyperopic shift at 2 months, followed by regression into a persistent myopic state and stabilized by the sixth month, indicating a delayed stabilization period compared to lower myopia groups.

### 3.2. The Related Factors Affecting the Final UCVA logMAR and UCVA Changes After Trans-PRK

The LMM analysis revealed that final corneal haze (*p* = 0.04) and final MSE (*p* < 0.001) emerged as the strongest drivers of final UCVA (Table 1). Furthermore, longer follow-up (*p* < 0.001) was associated with better visual outcome, likely reflecting long-term corneal remodeling. The final UCVA ≥ 0.8 accounted for 94.3% in the LM group, 95.3% in the MHM group, 88.8% in the EHM group, and 92.8% in the total sample.

**Figure 2:**
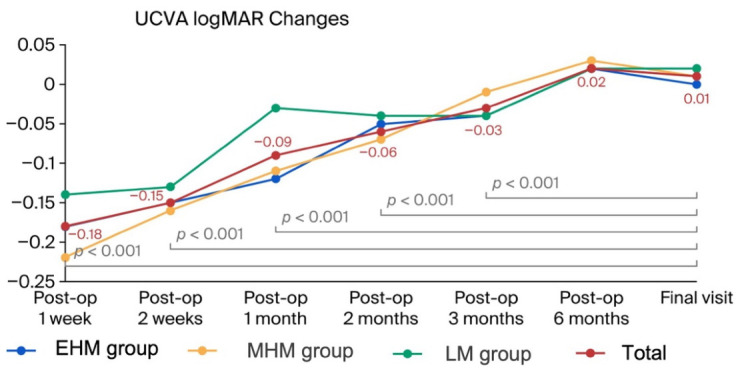
Overall, UCVA significantly improved following Trans-PRK, stabilizing by six months postoperatively. Recovery kinetics were stratified by myopia severity: the low myopia (LM) group showed biphasic gains (pre-1 month and months 2–3), while the moderate-to-high (MHM) group plateaued by month two. Conversely, the extremely high myopia (EHM) group exhibited a prolonged improvement trend, continuing through the full three-month duration.

Overall, mean UCVA significantly improved from −0.18 (SE, 132/200) ± 0.17 logMAR at 1 week to 0.01 (SE, 205/200) ± 0.08 logMAR at the final visit (*p* < 0.001), with continuous recovery observed throughout the first three months (Figure 2). Progressive visual recovery was evident across all three subgroups; in the LM group, vision improved from −0.14 (SE, 145/200) ± 0.15 logMAR (1 week) to 0.04 (SE, 218/200) ± 0.10 logMAR at the final visit, with significant gains through the third month (*p* < 0.001). In the MHM group, vision rose significantly from −0.22 (SE, 120/200) ± 0.17 logMAR (1 week) to −0.07 (SE, 170/200) ± 0.12 logMAR at two months (*p* = 0.003), stabilizing at 0.03 (SE, 214/200) ± 0.06 logMAR (6 months). In the EHM group, vision significantly improved from −0.18 (SE, 133/200) ± 0.17 logMAR (1 week) to −0.04 (SE, 182/200) ± 0.10 logMAR by three months (*p* < 0.001), reaching a final UCVA of 0.01 (SE, 205/200) ± 0.07 logMAR. Overall, UCVA stabilized by the sixth postoperative month across all myopia severities.

### 3.3. The Related Factors Affecting Corneal SPK and Their Presentation After Trans-PRK

Multivariate GEE analysis identified the discrepancy between measured and performed CCET (*B* = −0.22; OR = 0.806; 95% CI, 0.66–0.99; *p* = 0.042), laser application interval (*B* = −0.01; OR = 0.924; 95% CI, 0.86–1.00; *p* = 0.046) and follow-up duration (*p* = 0.031) as significant independent predictors of final corneal SPK (Table 1). Negative coefficients indicated that smaller CCET differences and shorter laser durations increased the risk of final corneal SPK. Ablation depth (*p* = 0.064) and MMC soaking duration (*B* = −0.16; OR = 0.851; 95% CI, 0.72–1.01; *p* = 0.068) fell short of statistical significance but were noted as borderline clinical trends. Preoperative myopia severity (*p* = 0.89) and refractive outcomes (*p* = 0.596) showed no significant association with SPK occurrence.

Of the twelve eyes (9.6%) that had corneal SPK at last visit, one eye (0.8%) was in the LM group with a mean follow-up of 6.26 ± 3.96 months, and five eyes (4%) were in the MHM group with a mean follow up of 6.02 ± 3.64 months while six eyes (4.8%) were in the EHM group with a mean follow-up of 8.05 ± 3.34 months.

**Figure 3:**
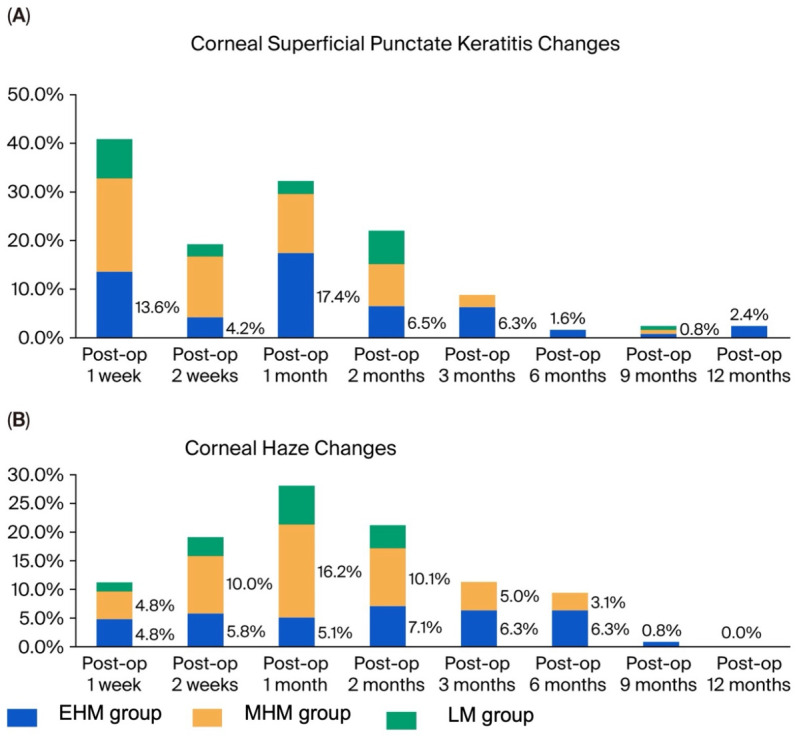
(**A**) Postoperative corneal SPK rates were 40.8%, 32.2%, 22%, 8.8%, and 1.6% at one week, one month, and two, three, and six months, respectively. Prevalence within the myopia subgroups peaked at one week [low myopia (LM): 8.0%; moderate-to-high myopia (MHM): 19.2%; extremely high myopia (EHM): 13.6%] before declining steadily. Notably, the LM group achieved complete resolution of SPK by two months postoperatively. While the MHM group reached zero prevalence by six months, the EHM group maintained a residual SPK rate of 1.6% at six-month follow-up. (**B**) The overall incidence of postoperative corneal haze peaked at 28.2% (LM: 6.8%; MHM: 16.2%; EHM: 5.1%) by the third month, followed by a gradual reduction to 9.4% (LM: 0.0%; MHM: 3.1%; EHM: 6.3%) at the six-month follow-up. Progression varied significantly by subgroup: the LM group achieved complete resolution (0.0%) by the third month, whereas the MHM group exhibited a steady decline through the sixth month. In contrast, the EHM group reached a plateau, maintaining a haze prevalence of approximately 6.3% at the six-month follow-up.

Postoperative corneal SPK prevalence peaked at one week (40.8%; LM: 8.0%, MHM: 19.2%, EHM: 13.6%) and remained at 32.2% at one month (LM: 2.6%, MHM: 12.2%, EHM: 17.4%). By six months, the LM and MHM groups achieved complete resolution, whereas a residual 1.6% prevalence persisted exclusively in the EHM group. Long-term follow-up (9–12 months) showed a stable residual rate of 2.4%, isolated primarily to EHM eyes (Figure 3A).

### 3.4. The Related Factors Affecting Corneal Haze and Its Presentation After Trans-PRK

Multivariate GEE analysis identified age (*B* = 0.31; OR = 1.37; 95% CI, 1.062–1.758; *p* = 0.015) and MMC soaking duration (*B* = 0.13; *OR* = 1.14; 95%CI, 1.01–1.29; *p* = 0.030) as the primary significant independent predictors of final corneal haze (Table 1). For every one-year increase in patient age, the odds of developing haze increased by 36.7% (*OR* = 1.37). Although not statistically significant, borderline clinical trends were noted for initial MSE (*p* = 0.055), ablation depth (*p* = 0.055), differences in measured and performed CCET (B = −0.267, *p* = 0.062), laser application interval (*p* = 0.064), follow-up duration (*p* = 0.063), and final UCVA logMAR (*p* = 0.051). Clinically, the presence of final corneal haze was associated with a +0.39 D hyperopia shift (haze/no haze= +0.23 D/−0.16 D), serving as a notable determinant of reduced final UCVA (*p* = 0.051) with a mean −0.03 (SE, 186/200) ± 0.02 logMAR [final UCVA of no haze: 0.01 (SE, 205/200)]. All cases were less than Grade 1, which did not interfere with the visibility of fine iris details [14].

Eleven eyes (8.8%) developed corneal haze by the final visit. These eyes had a mean relative decline in final UCVA of 0.04 (SE, 182/200) ± 0.06 logMAR compared with eyes without haze, a mean hyperopia shift in final SE of +0.23 D. In contrast, eyes without corneal haze demonstrated a mean final UCVA of 0.01 (SE, 205/200) ± 0.08 logMAR and a mean final MSE of −0.16 ± 0.74. Although patients with final corneal haze were univariately younger than those without (23.82 ± 5.51 vs. 30.82 ± 6.09 years), this trend was heavily confounded by the higher baseline myopia and greater ablation depths characteristic of our younger demographic, a factor accounted for in the final multivariate analysis. Among twelve eyes (9.6%) with final corneal haze, three eyes (2.4%) were in the LM group, and five eyes (4%) were in the MHM group, while three eyes (2.4%) were in the EHM group.

Postoperative corneal haze prevalence peaked at three months (28.2%; LM: 6.8%, MHM: 16.2%, EHM: 5.1%) before declining. By six months, prevalence decreased to 9.4%, with the LM group achieving complete resolution while the MHM (3.1%) and EHM (6.3%) groups showed residual traces. By nine months, total prevalence fell to 0.8%, isolated entirely to the EHM group. Overall, haze followed a transient course in the LM and MHM groups but persisted longer in EHM eyes (Figure 3B).

### 3.5. Differences Between the Groups of LM, MHM, and EHM

An LMM and a GEE were used to compare the three groups—LM (>−5 D), MHM (−5.0 D to −8.0 D), and EHM (≤−8 D)—as shown in Table 2. The LMM analysis identified deeper ablation thickness (*p* < 0.001), longer MMC soaking duration (*p* < 0.001), and laser application interval (*p* < 0.001) as the most critical predictors of final corneal haze and modulated haze risk. The LMM analysis also demonstrated no significant difference in final UCVA across the three initial myopia groups. Final UCVA remained stable (0.004 to 0.015 logMAR) with high pairwise consistency (all *p* > 0.89). These results confirm the visual safety and predictability of Trans-PRK across a broad refractive range. The LMM analysis revealed a significant refractive impact from initial MSE (*p* < 0.001), with the EHM group showing a slight myopic shift (−0.38 D; *p* = 0.0363) relative to the LM group. An intraclass correlation coefficient (ICC) of 0.532 validated the use of LMM to account for inter-eye correlation, ensuring statistical robustness in these bilateral outcomes. No significant difference in final corneal haze and SPK was observed.

### 3.6. Comparison of Various Refractions

We compared differences among several refraction types—initial manifest refraction, cycloplegic refraction, and subjective refraction with determined refraction—against the final manifest refractions in three groups (Table 3). The EHM group exhibited a slight myopic shift (mean: −0.38 ± 0.13 D), which differed significantly from the LM group (mean: +0.05 ± 0.13 D). The LM and EHM groups remained near emmetropia (mean: −0.05 ± 0.11 D).

Multivariate analysis (LMM) confirmed that subjective refraction was the strongest predictor for emmetropia in the LM and MHM groups, whereas initial MSE was most accurate for both LM and EHM groups. Cycloplegic refraction served as a consistent secondary predictor across all cohorts.

In summary, treatment planning based on cycloplegic and subjective refractions yields outcomes closest to emmetropia for LM/MHM Groups. Utilizing initial MSE refraction for the EHM group is recommended; though this targets a slight myopic shift, it achieves superior final emmetropic accuracy.

## 4. Discussion

Consistent with previous findings in the literature, refractive predictability at the 12-month follow-up is generally high across both myopic [15] and highly myopic [16,17,18] cohorts. In our current study, the proportion of eyes achieving a target refraction within ±0.50 D was fully comparable or superior to these published benchmarks across all myopic severity groups. Our study yielded comparable outcomes, with a mean final MSE of −0.12 D overall, and −0.38 D in the extreme high myopia (EHM) group. Predictability rates were 94.4%, 88.9%, and 94.3% for the final MS ≤ −1.0 D, final MSE ≤ −1.0 D, and UCVA ≥ 0.8, respectively. To achieve optimal emmetropic results, we evaluated the accuracy of various preoperative refractive metrics compared to the achieved postoperative outcomes. Our findings indicate that cycloplegic refraction was the most accurate predictor for both spherical power and MSE across the entire study population. This was followed closely by subjective refraction, which demonstrated high concordance with the final determined refraction. In contrast, initial manifest refraction was the least predictive metric. Notably, the EHM group consistently exhibited a greater degree of residual myopic shift relative to the MHM and LM groups across all refractive measures. Consequently, planning for EHM requires a systematic approach: first, prioritize residual stromal bed thickness of more than 300 µm to safeguard deep-ablation biomechanics; second, anchor laser programming on initial MSE and cycloplegic refractions—our strongest outcome predictors; and finally, apply a strategic mild overcorrection to counteract late-stage regression.

Multivariate analysis confirmed the high precision of Trans-PRK, showing that the overall post-operative mean MSE did not deviate significantly from emmetropia. The lack of significance during the follow-up periods further suggests that refractive outcomes remain stable over time once the initial healing phase is complete. While lacking statistical significance, this hyperopic shift is clinically relevant. Active collagen deposition and central subepithelial fibrosis flatten the anterior corneal curvature, reducing net refractive power to induce a hyperopic regression trend. Additionally, the interaction between final corneal SPK and final UCVA suggests that corneal surface regularity is a potential determinant of optimal functional outcomes, whereas final corneal SPK (*p* = 0.956) does not independently shift refractive power. MMC usage was not a significant predictor of final SE (*p* = 0.988). This indicates that while MMC is essential for haze prophylaxis, its application does not introduce refractive unpredictability, supporting its safety profile in surface ablation.

Furthermore, the factors associated with the final UCVA were the follow-up period, final corneal haze, and final MSE. Refractive stability near emmetropia is fundamental to visual success. In the LM and MHM groups, rapid MSE stabilization by the second week facilitated early visual recovery. In contrast, the EHM group maintained a slight hyperopia tendency (+0.32 D), contributing to a final UCVA of 18/20. Corneal haze peaked at three months (28.2%) but was primarily limited to Grade I opacification, which did not significantly hinder recovery. As haze declined to 9.4% by six months, a corresponding “jump” in visual acuity occurred. This suggests that while mild haze may cause transient fluctuations, it does not limit final visual potential. Once remodeling concludes, visual outcomes normalize across all groups, confirming that Trans-PRK-induced haze is a temporary phenomenon with no long-term adverse effect on functional vision.

Trans-PRK recovery trajectories vary significantly with myopia severity. The LM and MHM groups achieved rapid stability, with cylinder and MSE outcomes stabilizing between two weeks and three months. These cohorts exhibited a transient hyperopic shift before settling into a stable, slightly hyperopic state. This pattern mirrors the topographic findings from Yang et al. [19], where significant initial corneal curvature flattening after Trans-PRK was followed by a gradual steepening and peripheral epithelial thickening over the longitudinal follow-up period. In contrast, the EHM group followed a volatile course, with hyperopia peaking at one month, followed by significant myopic regression. Statistical stability was not reached until the third postoperative month. This delayed stabilization and higher residual cylinder likely reflect intensive epithelial remodeling and stromal healing following deeper ablations. While UCVA improved across all groups through six months, final visual outcomes remained superior in the LM and MHM cohorts. These findings suggest that extreme myopia requires a longer stabilization period and closer monitoring of the epithelial–stromal interaction to manage late-stage myopic shifts.

Bandeira et al. [20] reported that subbasal nerve regeneration reached 50% at 6–8 months and 90% by 2 years [21], while corneal sensitivity recovered to 80% at 1 week and nearly fully within 3–6 months [20,22,23]. However, reduced TBUT and Schirmer test values [22,23] and increased symptom scores [24] were noted at 1, 3, and 6 months post-PRK. Postoperative corneal SPK directly reflects secondary dry eye severity and corneal nerve regeneration, serving as a longitudinal surrogate marker for neurotrophic axis recovery. In our study, significant predictors of final corneal SPK included CCET discrepancy, laser interval, and follow-up duration. The role of follow-up (*p* = 0.031) confirms that surface and tear film stabilization is a progressive, long-term process. The significant negative correlation for CCET discrepancy (*B* = −0.22, *p* = 0.042) suggests that a thinner-than-planned epithelial profile leads to prolonged corneal SPK presence. Additionally, the negative coefficient for laser interval (*B* = −0.01, *p* = 0.046) indicates that shorter treatment durations were associated with an increased tendency toward postoperative corneal SPK development. Overall, corneal SPK after Trans-PRK decreased to less than 10% by three months postoperatively. The 3-to-6-month recovery window for corneal sensitivity post-Trans-PRK corresponds with literature reports of temporary tear film instability (reduced TBUT and Schirmer scores). This is strongly supported by our cohort’s data, where corneal SPK was largely cleared by 3 months.

Gadde et al. [25] observed a higher incidence of trace haze in single-step Trans-PRK than in manual PRK, particularly in eyes with higher myopia. This aligns with Møller-Pedersen et al. [26] and Spadea et al. [27], who demonstrated that haze severity and duration increase proportionally with ablation depth. Abdel-Radi et al. [28] observed corneal haze at 3 months postoperatively in approximately 40% of eyes across two-step and single-step Trans-PRK, which is substantially higher than our rate of 11%. In the present study, age and MMC soaking duration were identified as significant factors influencing final corneal haze. The significance of age suggests that older corneas may have an increased risk of subepithelial fibrosis after Trans-PRK. Furthermore, a higher initial MSE requires a deeper ablation depth [26,29], leading to extended laser intervals and longer MMC exposure, reflecting an “energy-response” relationship; however, this pharmacological modulation did not always completely suppress the proliferation and differentiation of limbal epithelial stem cells [30] in extreme myopia, a trend not observed in mild and moderate myopia [31]. Here, the negative correlation of the CCET discrepancy indicates that a thinner-than-planned epithelial profile impacts clinical healing dynamics, potentially contributing to postoperative corneal haze. Clinically, subepithelial fibrosis-induced haze served as a transient anatomical finding yet contributed to temporary refractive instability. This resulting hyperopic shift directly explains the reduction in final UCVA, highlighting the importance of haze prevention. In our cohort, all cases with final corneal haze maintained a mean final vision exceeding 18/20, with haze severity consistently below Grade I [13]. Corneal haze prevalence peaked at 1 month postoperatively and decreased thereafter, though it persisted beyond 6 months in the EHM group. These results are compatible with Lu et al., who demonstrated that intraoperative MMC application in Trans-PRK reduces haze incidence but has no long-term effect on epithelial remodeling [30], final visual and refractive outcome.

In high myopia, lamellar procedures like FS-LASIK (mean initial MSE: −7.50 ± 1.12 D) [32] and SMILE (mean initial MSE: −7.53 ± 1.18 D) [33] provide faster initial recovery but require deeper stromal cuts, increasing ectasia and regression. However, both conventional LASIK and FS-LASIK with concurrent cross-linking, which effectively mitigates ectasia risk, reported zero incidence of iatrogenic keratoconus. Conversely, our Trans-PRK cohort (achieved competitive long-term refractive stability despite a much higher refractive error in the EHM subgroup (mean initial MSE: −9.59 ± 0.15 D). While surface ablation historically risks deep-treatment haze, optimized spot geometry and intraoperative MMC effectively controlled tissue remodeling, yielding minimal final haze with no impact on final visual outcomes, confirming Trans-PRK as a safe, stable alternative. LMM analysis revealed that initial MSE significantly impacts final refractive outcomes (*p* = 0.047). While the LM and MHM groups achieved near-perfect emmetropia, the EHM group exhibited a slight myopic shift. Although this shift only approached significance compared to low myopia (*p* = 0.053), it suggests a trend toward regression in extreme cases. The ICC of 0.532 confirmed strong inter-eye correlation, validating the use of LMM. This study is limited by its retrospective design, bilateral eye inclusion, and variable follow-up periods (1–13.96 months), though this temporal variation minimally impacted our interpretations. To ensure statistical robustness, we employed an LMM and a GEE. This approach accounts for nested data, preventing the inflated Type I error rates typical of standard bilateral eye analysis. Additionally, longitudinal analysis showed that refractive stability, epithelial recovery, and haze regression plateaued at 6 months; thus, follow-up variances post-stabilization did not significantly bias the final clinical or safety outcomes.

## 5. Conclusions

TPRK demonstrates excellent efficacy and stability, with MSE and UCVA stabilizing by the third and sixth postoperative months, respectively. Multivariable analysis identified postoperative UCVA, corneal haze, and follow-up duration as primary determinants of refractive and functional outcomes. Additionally, corneal health—indicated by final corneal SPK and haze—was significantly influenced by ablation depth, laser duration, MMC soaking duration, and the discrepancy between measured and performed epithelial thickness. Subgroup analysis revealed that while the LM and MHM groups achieved near-perfect emmetropia and complete resolution of final corneal SPK and haze, the EHM group exhibited a distinct clinical profile. This cohort experienced a slight but significant myopic shift (−0.38 D) and maintained a residual haze prevalence of 6.3% at six months. These findings suggest that while Trans-PRK is effective across all severities, the EHM requires more intensive assessment according to the initial MSE or cycloplegic refraction to avoid late-stage regression and monitoring of the wound-healing response to manage persistent epithelial instability.

## Figures and Tables

**Table 1:** Baseline characteristics and associated factors of the study population (N = 125 eyes).

Mean (SD)/N (%)/*p*	Total (125 Eyes)	*p* Corneal SPK	*p* Corneal Haze	*p* Final SE	*p* Final UCVA logMAR
Age, y	30.20 (6.34)	0.977 ^b^	0.015 ^b^ *	0.780 ^a^	0.066 ^a^
Gender (M)	42 (33.60%)	0.849 ^b^	0.931 ^b^	0.002 ^a^ *	0.793 ^a^
OD	51 (50%)	0.104 ^b^	0.773 ^b^	0.538 ^a^	0.990 ^a^
**Preoperative factors**					
Initial manifest refraction: sphere power, D	−6.42 (2.27)	0.211 ^b^	0.193 ^b^	0.361 ^a^	0.362 ^a^
spherical equivalent, D	−7.16 (2.31)	0.890 ^b^	0.310 ^b^	0.110 ^a^	0.185 ^a^
CCET, µm	52.78 (4.19)	0.336 ^b^	0.454 ^b^	0.568 ^a^	0.380 ^a^
CCT, µm	531.27 (30.60)	0.682 ^b^	0.988 ^b^	0.011 ^a^ *	0.209 ^a^
**Operative factors**					
Determined refraction: sphere power, D	−6.01 (2.03)	0.850 ^b^	0.060 ^b^	0.005 ^a^ *	0.295 ^a^
spherical equivalent, D	−6.66 (2.05)	0.890 ^b^	0.055 ^b^	0.006 ^a^ *	0.373 ^a^
Ablation depth µm	164.34 (29.33)	0.064 ^b^	0.080 ^b^	0.228 ^a^	0.499 ^a^
Residual thickness, µm	366.90 (45.08)	0.075 ^b^	0.110 ^b^	0.123 ^a^	0.738 ^a^
Differences in measured and performed CCET, µm	−2.75 (3.07)	0.042 ^b^ *	0.062 ^b^	0.246 ^a^	0.642 ^a^
Ablation diameter, mm	6.48 (0.24)	0.090 ^b^	0.130 ^b^	0.671 ^a^	0.834 ^a^
MMC soaking duration, second	61.79(12.35)	0.068 ^b^	0.030 ^b^ *	0.532 ^a^	0.627 ^a^
Laser applied interval, second	57.56 (7.42)	0.046 ^b^ *	0.064 ^b^	0.274 ^a^ *	0.680 ^a^
**Postoperative factors**					
Follow-up periods, month	6.69 (3.73)	0.031 ^b^ *	0.063 ^b^	0.091 ^a^	<0.001 ^a^ *
Corneal SPK	12 (9.6%)		0.518 ^b^	0.704 ^a^	0.580 ^a^
Corneal haze	11 (8.8%)	0.518 ^b^		0.043 ^a^ *	0.040 ^a^ *
Final manifest refraction: sphere power, D	0.17 (0.71)	0.677 ^b^	0.451 ^b^		0.874 ^a^
spherical equivalent, D	−0.12 (0.73)	0.596 ^b^	0.834 ^b^		<0.001 ^a^ *
Final UCVA, logMAR	0.01 (0.08)	0.711 ^b^	0.051 ^b^	0.009 ^a^ *	

* *p* < 0.05, ^a^ Linear Mixed Model, ^b^ Generalized estimation equation; SD, standard deviation; N, number; %, percentage; D, diopter; CCET: central corneal epithelial thickness; CCT: central corneal thickness; SPK, superficial punctate keratitis; SE, spherical equivalent; UCVA: non-corrected visual acuity; D, diopter; y, year; M, male; logMAR, logarithm of minimum angle of resolution; Note: This table displays univariate correlations between baseline metrics and clinical outcomes (refractive, visual, haze, and SPK). Reading example: In the Age row (mean: 30.20 ± 6.34 years), horizontal tracking reveals a significant association with postoperative corneal haze (*p* = 0.015). Conversely, the initial manifest refraction: spherical equivalent row (mean: −7.16 ± 2.31 D) shows no significant relationship with postoperative SPK (*p* = 0.890), haze (*p* = 0.310), final SE (*p* = 0.110), or final UCVA (*p* = 0.185).

**Table 2:** The baseline characteristics and related factors of three groups, including low myopia (>−5.0 D), moderate-to high myopia (−5.0 D to −8.0 D), and extremely high myopia (≤−8.0 D).

Mean (SE)/N (%)/*p*	Extremely High Myopia (EHM) ≤−8.0 D (37 Eyes, 30%)	Moderate-to-High Myopia (MHM) −5.0 D to −8.0 D (53 Eyes, 42%)	Low Myopia (LM) >−5.0 D (35 Eyes, 28%)	*p*
Age, y	30.44 (0.89)	30.01 (0.86)	30.37 (0.88)	0.486 ^a^
Gender (M)	12 (32.40)	17 (32.10)	13 (37.1)	0.756 ^b^
OD	19 (51.40)	27 (50.90)	17 (48.6)	0.745 ^b^
**Preoperative factors**				
Initial manifest refraction: spherical power, D	−9.17 (0.15)	−6.28 (0.13)	−3.73 (0.15)	<0.001 ^a^ *
Spherical equivalent, D	−9.59 (0.20)	−7.09 (0.16)	−4.69 (0.20)	<0.001 ^a^ *
CCET, µm	51.47 (0.74)	3.15 (0.61)	53.45 (0.73)	0.104 ^a^
CCT, µm	536.44 (4.73)	523.56 (4.32)	525.51 (4.59)	0.016 ^a^ *
**Operative factors**				
Differences in measured and performed Corneal epithelial thickness, µm	−3.26 (0.54)	−2.43 (0.44)	−2.09 (0.53)	0.246 ^a^
Ablation depth, µm	182.67 (3.42)	165.36 (2.90)	141.67 (3.28)	<0.001 ^a^ *
Mitomycin-C soaking duration, second	59.52 (1.13)	57.92 (1.00)	55.22 (1.09)	<0.001 ^a^ *
Laser applied interval, second	67.90 (1.79)	61.72 (1.48)	54.77 (1.72)	<0.001 ^a^ *
**Postoperative factors**				
Follow-up periods, month	7.43 (0.54)	6.30 (0.49)	6.35 (0.53)	0.027 ^a^ *
Corneal SPK	6 (16.2)	5 (9.40)	3 (9.10)	0.173 ^b^
Corneal haze	3 (8.1)	5 (9.40)	2 (5.70)	0.795 ^b^
Final manifest refraction: sphere power, D	−0.07 (0.13)	0.23 (0.11)	0.33 (0.13)	0.052 ^a^
spherical equivalent, D	−0.38 (0.13)	−0.05 (0.11)	0.05 (0.13)	0.036 ^a^ *
Final UCVA, logMAR	0.01 (0.02)	0.01 (0.01)	0.00 (0.01)	0.896 ^a^

* *p* < 0.05, ^a^ Linear Mixed Models, ^b^ Generalized Estimating Equations; SE, standard error; N, number; %, percentage; CCET: central corneal epithelial thickness; CCT: central corneal thickness; SPK, superficial punctate keratitis; SE, spherical equivalent; UCVA: non-corrected visual acuity; D, diopter; y, year; M, male; logMAR, the logarithm of the minimum angle of resolution.

**Table 3:** Differences between all refractions, determined refractions, and final refractions.

Total: 64 Patients, 125 Eyes	Total (125 Eyes)	Extremely High Myopia (EHM) ≤−8.0 D (37 Eyes, 30%)	Moderate-to-High Myopia (MHM) −5.0 D to −8.0 D (53 Eyes, 42%)	Low Myopia (LM) >−5.0 D (35 Eyes, 28%)
Differences between initial manifest and determined refraction: Spherical equivalent, D LMM: mean, SE; *p* = 0.002 ^a^ *	0.67 (0.17)	−0.54 (0.11)	−0.26 (0.09)	0.03 (0.11)
Differences between cycloplegic and determined refraction: Spherical equivalent, D LMM: mean, SE; *p* = 0.286 ^a^	0.26 (0.19)	−0.02 (0.15)	−0.00 (0.09)	0.19 (0.10)
Differences between subjective and determined refraction: Spherical equivalent, D LMM: mean, SE; *p* = 0.491 ^a^	−0.35 (0.21)	0.03 (0.07)	−0.05 (0.06)	0.04 (0.07)
Final manifest refraction: Spherical equivalent, D LMM: mean, SE; *p* = 0.036 ^a^ *	−0.12 (0.08)	−0.38 (0.13)	−0.05 (0.11)	0.05 (0.13)

* *p* < 0.05; ^a^ LMM, Linear Mixed Models; SE, standard error; D, diopter.

## Data Availability

All data supporting this study will be shared upon request, although the majority are included within the manuscript.

## Abbreviations

PRK: photorefractive keratectomy; LASIK: laser in situ keratomileusis; SMILE: small incision lenticular extraction; FS-LASIK: femtosecond LASIK; Trans-PRK: transepithelial PRK; BCVA: best-corrected visual acuity; LM: low myopia; MHM: moderate-to-high myopia; EHM: extremely high myopia; UCVA: uncorrected visual acuity; MS: manifest sphere; MSE: manifest spherical equivalent; SE: Snellen equivalent; logMAR: the logarithm of the minimum angle of resolution; CCET: central corneal epithelial thickness; CCT: central corneal thickness; AS-OCT: anterior segment optical coherence tomography; SPK: superficial keratitis.
